# Comparative Analysis of Anaesthetic Efficacy of 2% Lignocaine With Dexmedetomidine as an Adjunct in Nerve Blocks for Dental Extractions: A Randomised Controlled Study

**DOI:** 10.7759/cureus.28867

**Published:** 2022-09-06

**Authors:** Tejas Suryawanshi, Anendd Jadhav, Aishwarya Gupta, Pooja Agrawal, Akhil Sharma

**Affiliations:** 1 Pedodontics and Preventive Dentistry, Sharad Pawar Dental College and Hospital, Wardha, IND; 2 Oral and Maxillofacial Surgery, Sharad Pawar Dental College and Hospital, Wardha, IND

**Keywords:** pain, orthodontic extraction, local anesthetic adjuvant, lignocaine, dexmedetomidine

## Abstract

Introduction

Adequate perioperative pain control through peripheral nerve blocks is a time-honored practice. Local anesthetic (LA) alone may fail to provide desirable pain control operatively. Dexmedetomidine (DEXMED), is a relatively latest addition to the class of α agonists. The present study was deliberated with the hypothesis that addition of DEXMED to LA does not alter the potency and efficacy of lignocaine. The primary outcome variable measured was pain. Onset, depth of anesthesia, and vital parameters duration of postoperative analgesia following administration of nerve blocks with the two solutions were also measured.

Method

A prospective, randomized, crossover, double-blind study was conducted on 60 systemically healthy subjects for extraction of premolars in all four quadrants. Subjects were randomly assigned to receive lignocaine mixed with epinephrine (2% lignocaine in 1:2,00,000 epinephrine) or lignocaine plus DEXMED (1μcg/ml lignocaine). On the second appointment of the study, the subjects received the other solution. Pulse rate, blood pressure, arterial oxygen saturation (SPo2), and respiratory rate were recorded as a baseline before performing, during, and two hours later.

Results

It showed the comparison of onset of anesthesia, and duration of anesthesia in between the two groups was found to be significant (p=0.00) in Group D and Group L. Number of subjects who consumed analgesics in Group L was 34 and in Group D was 14. The hemodynamic parameters displayed no statistically significant difference from their baseline values in the two groups.

Conclusion

The study concluded that dexmedetomidine when administered with lignocaine in nerve blocks provides greater hemodynamic stability and increases its anesthetic and analgesic potency making it a suitable addition to the existing list of additives for local anesthetic agents.

## Introduction

Local anesthetics (LA) are the basic tenets of pain control in dentistry. Pain control through peripheral nerve blocks with potent local anesthesia is a widely accepted and time-honored practice in oral surgery. LA alone may fail to provide desirable pain control peri-operatively due to its shorter duration of action compared to LA with adjuvants. Adjuvants added to LA may enhance its potency, and efficacy and prolong the duration of nerve blocks. The various agents used as adjuvants with local anesthetics are vasoconstrictors like epinephrine, opioid, steroids such as dexamethasone, non-opioid analgesics, and α2 receptor agonists.

Lignocaine is considered a gold standard LA agent against which others are compared. Epinephrine is the most common and widely used additive with lignocaine, epinephrine acts on α and predominantly on β adreno-receptors. The anesthetic solution with a combination of lignocaine and epinephrine has been consistently found to confer superior results when compared to plain (lignocaine without epinephrine) anesthetic solution [1,2]. However, epinephrine has pronounced, undesirable cardiovascular effects and hence it is advised to be used with attentiveness in patients with cardiovascular and circulatory disorders. Continued quest of finding an ideal additive led researchers to use dexmedetomidine (DEXMED) which is a relatively newer addition to the class of α agonists. Dexmedetomidine has a greater affinity for α2 receptors and offers greater hemodynamic stability. Dexmedetomidine acts by binding to pre-synaptic receptors, resulting in restrains of the secretion of epinephrine, thus preventing the propagation of pain impulses [3,4].

Khandaitkar et al. [5] conducted a study on 90 subjects to assess the efficacy of dexmedetomidine with 2% lignocaine on infraorbital nerve block in three groups. The first group (control) was given 2% lidocaine 2 ml only, the second, 2% lidocaine 2 ml with dexmedetomidine 14g peripherally (peripheral group), and the third, 2% lidocaine 2 ml peripherally with dexmedetomidine 14g systemically (systemic group). The onset of anesthesia, duration of action, blood pressure, oxygen saturation, and heart rate were evaluated. Results showed that onset and duration of anesthesia were faster and prolonged in the peripheral group as compared to other groups with insignificant hemodynamic changes amongst the groups. This study concluded that the addition of 7 mg/L dexmedetomidine to lidocaine perineurally boosts the onset of action and prolongs the duration of anesthesia [6-10].

Limited studies have evaluated the perineural use of dexmedetomidine with lignocaine in dentistry. Thus, the present study was deliberated with the premise that the addition of dexmedetomidine into local anesthetic does not affect the potency and efficacy of lignocaine with the hypothesis that dexmedetomidine provides better analgesic and anesthetic potency as a local anesthetic adjunct. The onset of anesthesia, depth of anesthesia, duration of postoperative analgesia, the total number of analgesics consumed, and the vital parameters are the variables constituted in the study.

## Materials and methods

The present prospective, randomized controlled, crossover, double-blind study was conducted on 50 systemically healthy subjects reporting to the outpatient department of Oral and Maxillofacial Surgery at Sharad Pawar Dental College and Hospital, Wardha for extraction of premolars in all four quadrants between August and September 2019. The study was conducted after the approval institutional ethical committee (Ref. no. DMIMS (DU)/IEC/2019/7989) and was performed conforming with the Standard Institutional Ethical guidelines prescribed by the Central Ethics Committee on Human Research (CECHR) of Datta Meghe Institute of Medical Science.

Fifty healthy subjects of Grade 1 American Society of Anesthesiologists (ASA) status, aged between 18-40 years with bilateral asymptomatic premolars indicated for extraction were included in the study. Subjects with systemic illness, pregnant women, nursing mothers, subjects on anti-hypertensive medications, chronic smokers, drug abusers, and subjects having allergies to the drug used in the study were excluded from the study. Also, subjects having failed anesthesia and subjects experiencing any untoward complications such as hematoma, ocular or facial nerve palsy were also excluded from the study. All the subjects were randomly allocated to Group L and Group D by using the computer generated table of random numbers. 

**Figure 1:**
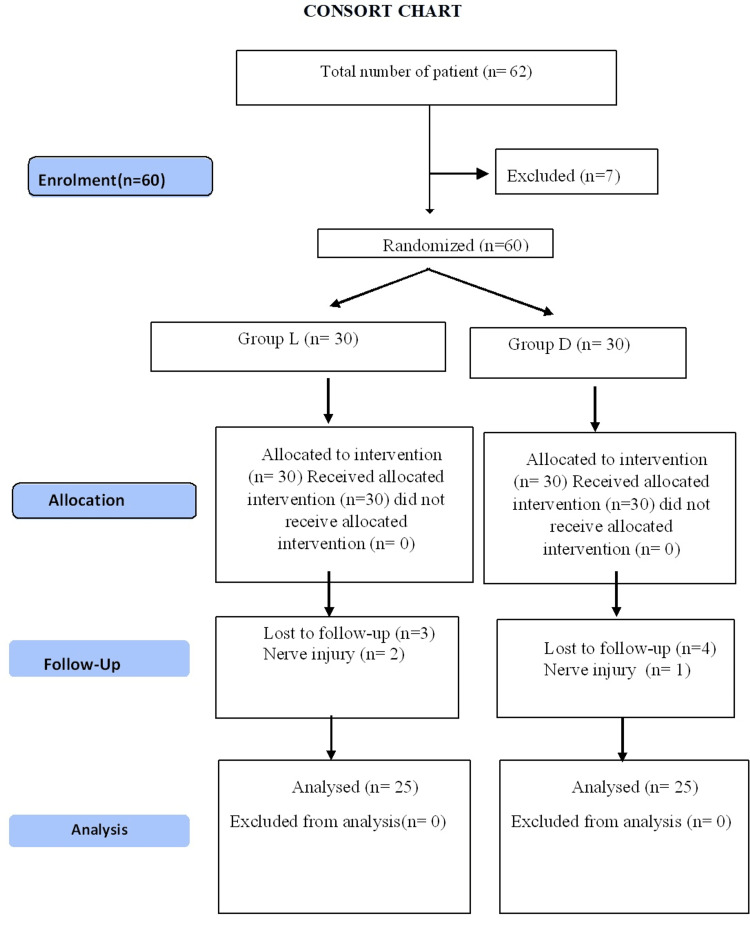
CONSORT Chart

The study was performed according to CONSORT (CONsolidated Standards of Reporting Trials) [11] guidelines for randomized control trials (Figure 1).

After obtaining written informed consent a thorough clinical evaluation may be performed. A split-mouth or crossover study design was implemented and each patient was subjected to the intra-alveolar technique in either jaw on both sides. Each subject was elucidated in depth about the study procedure and the visual analog scale. Subjects were studied on two separate days and the washout period between two interventions (homolateral and contralateral side) was four days (96 hours).

On the first appointment, subjects were randomly assigned to receive lignocaine mixed with adrenaline (2% lignocaine in 1:2,00,000 adrenaline) or lignocaine and dexmedetomidine (30μgm in 30 ml lignocaine, constituting 1μcg/ml DEXMED). The side of the administration of epinephrine as an additive was connoted as Group L, which served as control whilst the other side where DEXMED was used acted as the study group was designated as Group D.

The test solution was arbitrarily assigned grounded on sequentially numbered opaque envelopes containing computer-generated codes. On the second appointment of the study, subjects were administered the other solution in a cross-over and double blinded technique (neither the subject nor the investigator was aware of the solution being injected on two consecutive appointments). At each appointment, a local anesthetic solution was administered via infraorbital nerve block 1.5ml and greater palatine nerve block for extraction of maxillary premolar tailed by mandibular premolars anesthetic solution delivery of 0.5ml of solution for lingual nerve block and 1.5ml of solution for inferior alveolar nerve block.

Subjects were asked to be seated comfortably in a semi-slanting position in the dental chair and monitoring devices were attached. Heart rate (HR), blood pressure (BP), arterial oxygen saturation (SPO2), and respiratory rate (RR) are taken as a baseline prior to performing extraction, during the extraction, and after 120 minutes post-operatively. The local anesthetic agent was administered. Any SPO2 values < 95% as well as any episodes of apnoea persisting for more than 20 seconds were recorded. No antibiotics were prescribed to the subjects. The analgesic used in the study was tab ketorolac tromethamine 10 mg (Torrodent DT, Cipla) one tab when and as required.

**Figure 2:**
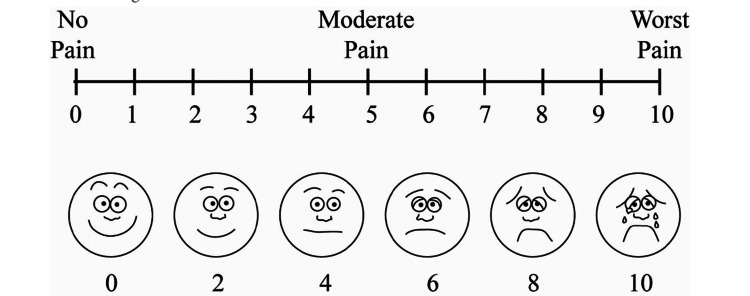
Visual analogue scale

The time of onset of action of anesthesia was assessed from the time of injection of the local anesthetic agent until complete loss of sensation is attained over the buccal gingiva of the subjected tooth to be extracted. The period of anesthesia was measured from the time of achievement of comprehensive loss of sensation over the prescribed area, until the time the subject requests for first rescue analgesic medication. The duration of postoperative analgesia was defined as “The time recorded from the point of completion of the extraction to the time when the pain intensity was scored ≥ 3 on visual analog scale (VAS) (Figure 2) or when the patient feels the need of consuming rescue analgesia.”

The time and number of consumption of the first and subsequent rescue analgesics were asked to be recorded and collected upon the completion of the study protocol from the patients.

The depth of anesthesia was assessed objectively by probing over the palatal and buccal soft tissue of the premolar to be extracted and was recorded on a visual assessment scale at 10 minutes of injection and then at intervals of 20 minutes, six times. The subjects were asked to mark their response on 10-point VAS scale each time after administration of LA. All the extractions were undertaken only after ascertaining the adequacy of anesthesia. Subjects were given recall after three days from the extraction of teeth of the contralateral side, abiding with the study protocol. As per the protocol, anytime the patient’s heart rate would drop below 50 beats/minute, intravenous inj. atropine 0.6mg would be administered immediately. The subjects were contacted and interrogated by the senior investigator on completion of the first post-operative 24 hours of surgery to learn the need and time of first analgesic taken.

Statistical analysis was done by using descriptive and inferential statistics. Qualitative analysis was done using chi-square test, whereas quantative analysis was done using Student’s paired and unpaired t test. Software used in the analysis was Statistical Package for Social Sciences (SPSS) version 24.0 (IBM Corp., Armonk, NY, USA) and Graph Pad Prism 7.0 version and p<0.05 was considered as level of significance.

## Results

The Group D exhibited a shorter onset of anesthesia for both maxilla and mandible (119.24 + 13.57 secs and 122.27 + 12.60 secs respectively) compared to Group L (172.72 + 14.94 secs for maxilla and 174.75 + 12.59 sec for mandible). The mean duration of onset of anesthesia (secs) between the two groups varied significantly (p=0.00) as shown in Table 1.

**Table 1:** Comparison of onset of anaesthesia between the two groups

TIME OF ONSET	DEXMED	ADRENALINE
MAXILLA	119.24±13.57 secs	172.72±14.94 secs
MANDIBLE	122.27±12.60 secs	174.75±12.59 secs
p- value	0.00 S	

No statistically significant results were found from their baseline values in the two groups in respect of heart rate, oxygen saturation, respiratory rate, and systolic and diastolic blood pressure as seen in Table 2.

**Table 2:** Comparison of vital parameters between the two groups RR: respiratory rate, HR: heart rate, SPO2: arterial oxygen saturation

Sr.No	PRE-OPERATIVE		INTRA-OPERATIVE		POST-OPERATIVE	
	GROUP D	GROUP L	GROUP D	GROUP L	GROUP D	GROUP L
1. MEAN SYSTOLIC	121.04±6.8956	119.04±8.2138	111.80±7.8584	112.32±7.1412	115.12±7.7949	119.04±8.8639
p value	0.190NS		0.730NS		0.21NS	
2. MEAN DIASTOLIC	80.44±6.0107	78.88±7.2720	72.24±6.0154	73.28±5.9489	78.04±8.0962	78.04±8.0964
p value	0.245 NS		0.387 NS		1.00 NS	
3. MEAN RR	17.86±1.5519	17.54±1.1381	20.88±1.2229	20.76±1.2047	19.00±1.7728	18.52±1.6566
p- value	0.280 NS		0.622 NS		0.165 NS	
4. MEAN HR	81.56±9.5728	81.24±9.6712	81.24±9.6712	81.46±9.6536	79.92±6.7848	79.96±6.7858
p-value	0.868 NS		0.910 NS		1.00 NS	
5. MEAN SPO_2_	100±0.0000	100±0.0000	100±0.0000	100±0.0000	100±0.0000	100±0.0000
p-value	1.00 NS		1.00 NS		1.00 NS	

Group D exhibited a longer duration of anesthesia for both maxilla and mandible (443.60 + 99.28 minutes and 446.12 + 86.32 minutes respectively) compared to Group L with a duration of anesthesia of 332.50 + 73.20 minutes for maxilla and 335.13 + 69.82 minutes for the mandible. The mean duration of anesthesia between the two groups varied significantly (p=0.00), as displayed in Table 3.

**Table 3:** Comparison of duration of anaesthesia between the two groups

DURATION	DEXMED	ADRENALINE
MAXILLA	443.60±99.28 mins	332.50±73.20 mins
MANDIBLE	446.12±86.32 mins	335.13±69.82 min
p value	0.00 S	

Subjects of Group D (14 subjects consumed 1 tablet each) consumed less analgesics compared to subjects of Group L (34 subjects consumed 1 tablet each) within 24 hours after extraction, signifying the analgesic-sparing effect of DEXMED. Statistically significant difference (p=0.00) existed between the two groups, as shown in Table 4.

**Table 4:** Comparing number of subjects consuming analgesic on post-operative day one between two groups

GROUP D	GROUP L
14	34

## Discussion

Adequate control of postoperative pain is a major concern as it is one of the major determinants of the success of the procedure. Good postoperative pain control leads to significant overall comfort, less analgesic dependence, early return to function and helps improve the overall quality of life. The quest for a suitable pharmacotherapeutic agent to maximize the analgesic potency without undermining the safety profile is interminable.

Studies reporting the efficacy of DEXMED as an adjunct in enhancing the efficacy of local anesthetic solution in peripheral nerve blocks are limited. Ask et al. observed similar potency of supraclavicular brachial plexus blockade when 15 ml of DEXMED (1µgm/ml) was added to 15 ml of 0.33% bupivacaine compared to 30 ml of 0.33% bupivacaine alone, implying local anesthetic sparing effect of DEXMED [7]. Similarly, in a recent study by Vallapu et al. a prolonged analgesia following a craniotomy procedure under regional scalp anesthesia was observed when DEXMED in the dose of 1µgm/ml was added to bupivacaine compared to bupivacaine alone [9]. Virendra et al. observed that the addition of DEXMED at 1µgm/ml to 2% lignocaine for maxillary and mandibular nerve blocks noticeably yielded a prolonged duration of anesthesia, fastened onset, and demonstrated improved post-operative analgesic requirements with no report of adverse events [6]. The preceding published results led the investigators to deliberate the present study to evaluate the efficacy, potency, and safety of DEXMED as an addition to 2% lignocaine by evaluating its time of onset, depth of anesthesia, the potency of postoperative analgesia, and any associated adverse events. The current study was instigated with the hypothesis that the addition of DEXMED to lignocaine does not affect the efficacy as well as the potency of local anesthetic agents.

Analysis of the data shows that the subjects of Group D demonstrated a shorter onset time and prolonged duration of anesthesia in the maxilla as well as mandible compared to Group L with significant differences (p<0.001 S). The depth of anesthesia of both the solution was found to be equipotent with insignificant differences (p>1.00 NS). These findings are by a study by Singh et al. [8]. The effect of DEXMED can be attributed to its local vaso-constrictive and anti-inflammatory effects; these effects may be responsible for prolonged anesthesia and good analgesia post-operatively [12-15]. Yamane et al. [4] also found similar results when dexmedetomidine was injected locally in the oral mucosa. The exact mechanism of action is unclear; however, it has been demonstrated that α2 receptor agonists have a direct inhibitory effect on the C-fiber action potential and sodium channels. It is also known to inhibit the release of substance P at the dorsal root neurons by activation of α2 receptors, thereby, inhibiting the nociceptive pathways.

The hemodynamic parameters such as HR, BP, RR, and O2 saturation displayed no statistically significant difference from their baseline values in the two groups. This implies that hemodynamic stability afforded is a desirable and additive contribution of DEXMED. None of the subjects in Group D demonstrated the need of abandoning the intervention or seeking urgent medical assistance. The intravenous route of administration of DEXMED with a dose of > 3µgm/kg has been shown to result in bradycardia, hypotension, and bradypnea [3].

Postoperative pain control is formidably concerned with the quality of intra- and postoperative anesthesia. The time of onset of pain and duration of anesthesia are not comparable as the evaluated pain is an individual entity and did not associate with other perceptions such as proprioception, temperature, and pressure. The duration of postoperative analgesia was defined as “The time recorded from the point of completion of the extraction to the time when the pain intensity was scored ≥ 3 on VAS or when the patient feels the need of consuming rescue analgesia” [14]. In the present study, the duration of analgesia (time taken from consumption of rescue analgesia) varied considerably between the two groups.

It was found to be 332.50 ± 73.20 mins in the maxilla and 335.13 ± 69.82 mins in the mandible of Group L and 443.60 ± 99.28 mins in the maxilla and 446.12 ± 86.32 mins in the mandible of Group D with a statistically significant difference (p=0.000 S).

The cumulative success rate of postoperative analgesia for both groups was evaluated based on the mean of analgesic dosages required. The number of subjects requiring analgesics in the first 24 hours was higher in Group L as compared to Group D. The number of subjects taking analgesics in Group L was 34, whereas in Group D 17 subjects took analgesics on postoperative day one, showing statistically significant results exhibiting prolonged duration of action and signifying the analgesic sparing action of DEXMED.

The authors of the present study found that the addition of DEXMED to lignocaine demonstrated shorter onset, prolonged duration of anesthesia, and good postoperative pain control with better hemodynamic stability and negligible adverse events. These aforementioned desirable properties may help add DEXMED to the existing list of additives to be used with local anesthetics. The present study was conducted upon young and healthy individuals and hence cannot comment on the safety of administration of DEXMED in patients with cardiorespiratory compromised status. The addition of DEXMED to lignocaine is a relatively novel concept and the present study may help instigate new avenues of research for newer adjuvants to be used with local anesthetic solutions. The present study has some inherent limitations such as a limited sample size and the lack of ability of the study design to comment on the safety profile for DEXMED in patients with compromised cardiorespiratory status.

## Conclusions

DEXMED when administered with lignocaine perineurally in maxillary and mandibular nerve blocks significantly prolongs its anesthetic efficacy and analgesic potency and affords greater hemodynamic stability making it a suitable addition to the existing list of additives for local anesthetic agents. This study may have the potential to overlay the conventional practice of using lignocaine in dentistry with the advanced effects of DEXMED.
